# Collagen Type II—Chitosan Interactions as Dependent on Hydroxylation and Acetylation Inferred from Molecular Dynamics Simulations

**DOI:** 10.3390/molecules28010154

**Published:** 2022-12-24

**Authors:** Maciej Przybyłek, Piotr Bełdowski, Florian Wieland, Piotr Cysewski, Alina Sionkowska

**Affiliations:** 1 Department of Physical Chemistry, Pharmacy Faculty, Collegium Medicum of Bydgoszcz, Nicolaus Copernicus University in Toruń, Kurpińskiego 5, 85-950 Bydgoszcz, Poland; 2 Institute of Mathematics and Physics, Bydgoszcz University of Science and Technology, al. Kaliskiego 7, 85-796 Bydgoszcz, Poland; 3 Helmholtz-Zentrum Hereon, Institute for Metallic Biomaterials, Max-Planck-Straße 1, 21502 Geesthacht, Germany; 4 Department of Biomaterials and Cosmetics Chemistry, Faculty of Chemistry, Nicolaus Copernicus University in Toruń, Gagarin 7, 87-100 Toruń, Poland

**Keywords:** chitosan, collagen, intermolecular interactions, affinity, binding, molecular dynamics

## Abstract

Chitosan–collagen blends have been widely applied in tissue engineering, joints diseases treatment, and many other biomedical fields. Understanding the affinity between chitosan and collagen type II is particularly relevant in the context of mechanical properties modulation, which is closely associated with designing biomaterials suitable for cartilage and synovial fluid regeneration. However, many structural features influence chitosan’s affinity for collagen. One of the most important ones is the deacetylation degree (DD) in chitosan and the hydroxylation degree (HD) of proline (PRO) moieties in collagen. In this paper, combinations of both factors were analyzed using a very efficient molecular dynamics approach. It was found that DD and HD modifications significantly affect the structural features of the complex related to considered types of interactions, namely hydrogen bonds, hydrophobic, and ionic contacts. In the case of hydrogen bonds both direct and indirect (water bridges) contacts were examined. In case of the most collagen analogues, a very good correlation between binding free energy and DD was observed.

## 1. Introduction

The proper function of joints depends on the optimal composition of synovial fluid and cartilage, which guarantees an effective biolubrication. Collagen and hyaluronan are probably the most important extracellular matrix molecules. Inter- and intramolecular interactions involving biomacromolecules in connective soft tissues significantly affect their nanomechanical features [1]. Collagen–hyaluronate mixtures are commonly used in various fields, including tissue engineering, pharmacy, and cosmetics [2,3,4]. An interesting, much cheaper, and easily available alternative for hyaluronan is chitosan, which shows quite similar features. However, there are also some differences. For instance, hyaluronan is in general more hydrophilic than chitosan, on the other hand, chitosan is more prone to aggregation than hyaluronan [5]. Due to its high biocompatibility and other unique features, collagen/chitosan blends were widely applied in tissue engineering, including vascular tissue-, skin-, bones- and cartilage-mimicking materials [6,7,8,9,10,11,12,13,14,15,16]. The chitosan precursor material namely chitin which contains the maximal number of acetyl groups can be easily obtained from various natural sources. This important biomaterial occurs in mushrooms, shrimps, crabs, silkworms, fungi, and green algae [17,18,19,20,21]. For this reason, chitin is considered a “green material”, since many of the above-mentioned sources such as shells of crustaceans are waste products. The chitin/chitosan carbohydrate chain consists of glucosamine (2-amino-2-deoxy-β-D-glucan) and N-acetylated glucosamine moieties linked by 1→4 glycosidic bonds. The number of acetyl groups in chitosan affects various features such as membrane permeability, viscosity, hydrophobicity, air/water contact angle, bio-compatibility, and solubility [22,23,24,25,26]. 

The affinity of chitosan to collagen is a crucial factor affecting important properties of biocomposites consisting of these macromolecules. This was confirmed by different studies that probed the intermolecular interactions between chitosan and collagen by combining various measurements to characterize the rheological, mechanical, and stability properties [27,28,29]. However, there is not much information in the literature on the influence of the deacetylation degree of chitosan and the degree of hydroxylation in type II collagen on the properties of composites. As was established by Hsu et al. [30] in the case of the alginate blends with chitosan, the low number of acetyl groups in chitosan is beneficial for the mechanical properties. This can be explained by the efficient assembly of biopolymers, caused by the high number of amino groups able to interact more strongly with the alginate than acetyl groups [30]. 

Proline (PRO) hydroxylation degree (HD) is an important factor affecting collagen properties, including mechanical behavior [31] and thermal stability [32]. The larger number of the hydroxyproline (HYP) residues, the higher the denaturation temperature. Importantly, HD seems to be also a crucial factor that may affect the stability of molecular complexes with chitosan. This hypothesis is based on several observations. For instance, according to Sipila et al. [33], the HYP/PRO ratio in collagen significantly affects the binding affinity toward integrin. However, collagen–chitosan systems have not been studied in terms of HD effect on complex stability. 

**Figure 1:**
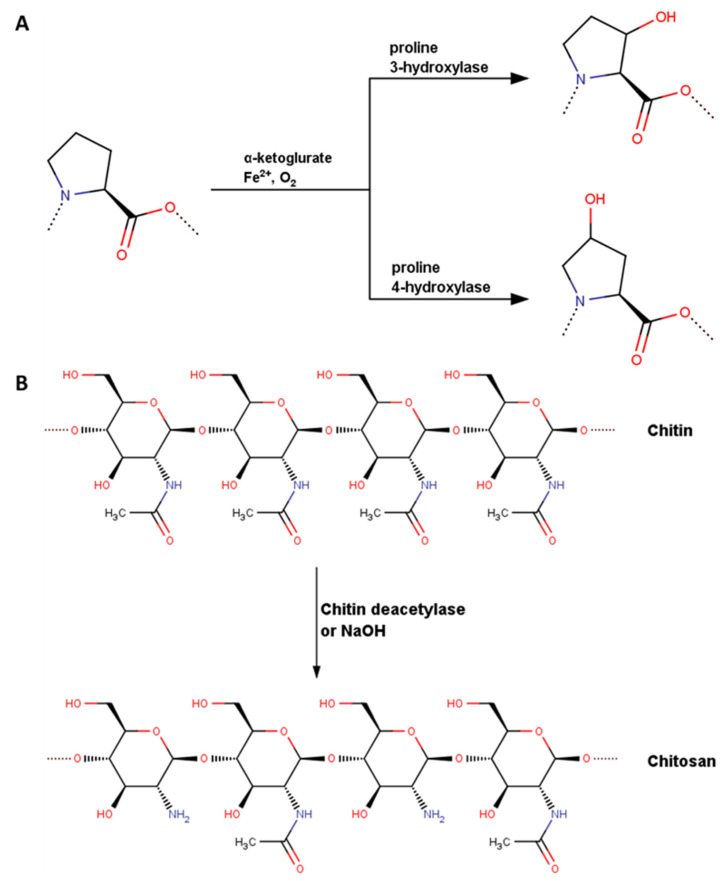
The enzymatic hydroxylation of the PRO moiety in collagen (**A**) and deacetylation of chitosan (**B**). In case of the reaction scheme shown on the panel A, L-proline moiety is hydroxylated, which is a natural isomeric form in collagen. In the current study the hydroxylation of the same PRO form yielding trans-4-Hydroxy-L-proline residues was taken into account.

The general scheme of chitin deacetylation and collagen PRO hydroxylation was presented in Figure 1. In the case of deacetylation, it can be carried out either by enzymatic process [34,35,36] or by using simple basic hydrolysis [26,37,38,39]. The former method is very limited and cannot be used to remove the majority of acetyl groups [36]. On the other hand, by using the latter method very high or even complete deacetylation can be achieved [38], however the process often leads to polymer chain degradation [26]. The PRO hydroxylation process in collagen analogues should be rather considered as HYP/PRO regulation by modulating the prolyl hydroxylase activity [33,40,41,42]. An experimental study that systematically investigates this problem is hard to conduct due to different limitations. An alternative to gaining deep knowledge is the use of molecular dynamics simulations to study all effects on various combinations of DD in chitosan and HD in collagen. Herein, the main aim of the research was to investigate how structural alterations in biomolecular chains affect the stability of the complexes.

## 2. Results and Discussion

**Figure 2:**
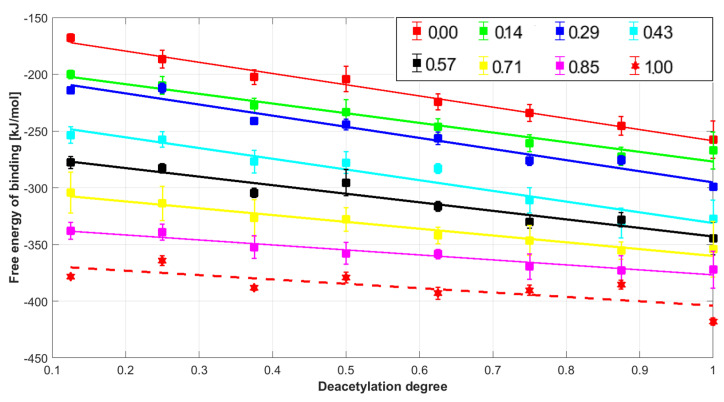
Free energy of chitosan-collagen binding as a function of both chitosan deacetylation degree (DD) and the hydroxylation degree HD.

**Figure 3:**
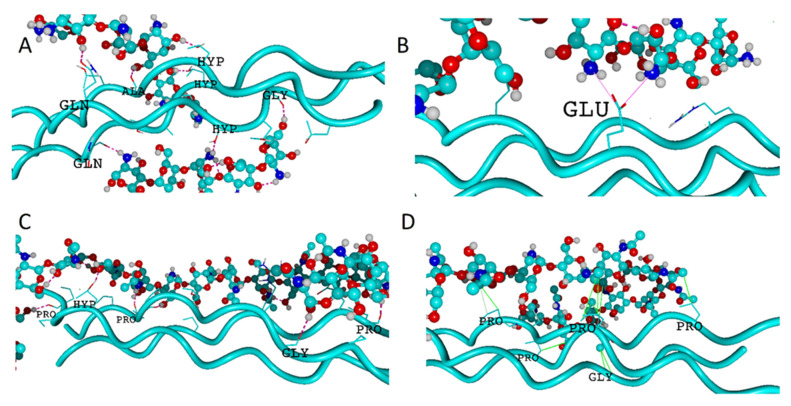
Visualization of collagen type II helices interacting with chitosan. The panels (**A**–**D**) represent exemplary snapshoots of the complexes characterized by HD = 1/DD = 1, HD = 0.5/DD = 1, HD = 0.5/DD = 0.5, and HD = 0/DD = 0.125, respectively. Hydrogen bonds were denoted by pink dotted lines, while ionic contacts by solid pink lines. Solid green lines stand for hydrophobic contacts. Collagen helices are presented as solid turquoise lines, whereas chitosan is represented by ball-stick model with colors representing: carbon-turquoise, nitrogen-dark blue, oxygen-red, and hydrogen-white.

In this study, the intermolecular interactions between collagen type II characterized by different HD degrees and chitosan molecules modified by alternating the number of acetyl moieties attached to the amino groups were examined. The analysis of Gibbs free energy values indicates that the formation of all complexes is thermodynamically favorable (Figure 2). The Gibbs free energy of binding is lower than −150 kJ/mol. Further, the stability of the collagen-chitosan complex is significantly affected by both HD and DD, as documented in Figure 2. All collagen structures with a different HD degree show a linear decrease in the Gibbs free energy as the DD content rises. The correlation coefficients R^2^ were 0.99 (HD = 0), 0.96 (HD = 0.14), 0.96 (HD = 0.29), 0.93 (HD = 0.43), 0.94 (HD = 0.57), 0.96 (HD = 0.71), 0.94 (HD = 0.85), and 0.58 (HD = 1.00). The increase in HD influences the binding affinity irrespectively of DD values. The highest affinity of chitosan to collagen was observed in the case of the completely hydroxylated and completely deacylated species (HD = 1 and DD = 1) molecular assembly. It is worth mentioning that the collagen characterized by the high HYP/PRO ratio is in general more stable [32]. Therefore, the high number of HYP residues promotes self-association during the gelling process [43,44]. According to some suggestions [45], this factor may have the effect of reducing the ability of collagen to complex chitosan, which of course is based on the supposition that the complexation takes place as a result of tertiary structure degradation. This behavior seems to be opposed when considering the use of chitosan as a substitute for hyaluronic acid. Nevertheless, it is known that this additive improves the thermal stability of collagen by increasing the denaturation temperature [46]. Thus, the interactions occurring on the collagen surface seem to be more favorable and maintain the consistency of the tertiary protein structure. Indeed, as one can infer from Figure 3, only the side functional groups are involved in the interactions between chitosan and collagen. This is consistent with the study of Taravel and Domard [47] showing that the calf skin collagen’s triple helix system stability is enhanced as a result of complexation with chitosan. On the other hand, some experimental results show that chitosan can affect the tertiary structure [27]. 

The high affinity of highly deacetylated chitosan can be explained by the significantly higher basicity of the amino group, which is more capable of interacting with carboxylic and hydroxyl groups. The high stability of the complexes formed by highly deacetylated chitosan HD > 75% and collagen was reflected by the exceptionally high mechanical durability of the composites [48,49,50]. Furthermore, highly deacetylated chitosan (c.a. 90%) is characterised by excellent miscibility with collagen [51]. Chitin (DD = 0) can also form blends with collagen [52]. This is consistent with the negative sign of free energy of binding, which can be noticed in the case of all chitin complexes (Figure 2). 

The graphical representation of the exemplary complexes was presented in Figure 3. As one can see the several types of intermolecular interactions (hydrogen bonds, ionic contacts, water bridges, and hydrophobic interactions) can be distinguished in the case of collagen–chitosan complexes.

### 2.1. Hydrogen Bonds

According to the default definition implemented in the YASARA force field [53], the energy of a particular contact can be determined from the atom distances, appropriate donor–hydrogen–acceptor, hydrogen–acceptor–X angle, and scaling factors by using the following Equation (1):

(1) $$E=-25\cdot \frac{2.6-\max\left(Dis_{H-A,}2.1\right)}{0.5}\cdot Scale_{D-H-A}\cdot Scale_{H-A-X}$$

In the case of *E* values higher than −6.25 kJ/mol, the interaction is classified as a hydrogen bond. Some structural details of studied molecular systems including characteristics of identified H-bonds was provided in Table S1–S25. Among all collagen–chitosan complexes considered in this study the most stable hydrogen bond was formed by GLU and chitosan O3 hydroxyl group (atoms numbering according to Supplementary Figure S1) in case of a structures characterized by DD = 1 and HD = 1 and the energy of this interaction was 25 kJ/mol. This contact was characterized by one of the shortest H-bond lengths (1.64 Å). The weakest H-bonds characterized by slightly higher energy than the threshold were ALA(N) and amine group in chitosan (E = −7.3 kJ/mol) for the same complex. In the case of collagen–chitosan complexes the most frequently appearing hydrogen bonds are formed by HYP, PRO, GLY, and residues, i.e., the most abundant amino acids in the structures. Chitosan residues can take both hydrogen bond accepting or donating parts. By comparing the three different variants of collagen HD = 0, HD = 0.42 (native) and HD = 1 some interesting differences in the H-bonds accepting roles can be observed (supplementary Tables S20–S22). First of all, the accepting role of ARG is increasing with the increase in HD. This can be explained by a closer distance of chitosan in the complex being the consequence of intermolecular interactions involving HYP residues. As expected also the DD affects the accepting role of the residues. For instance, when considering the complexes involving the native collagen, the number of H-bonds formed by ARG playing the accepting role is higher for DD = 0.5 (Table S24) than for DD = 1 (Table S21). These observations are intuitive and show an increase in the number of basic centers as a consequence of deacetylation, which affects the accepting abilities. The consequence of this is, in turn, a reduction in the general accepting role of the collagen residues. 

**Figure 4:**
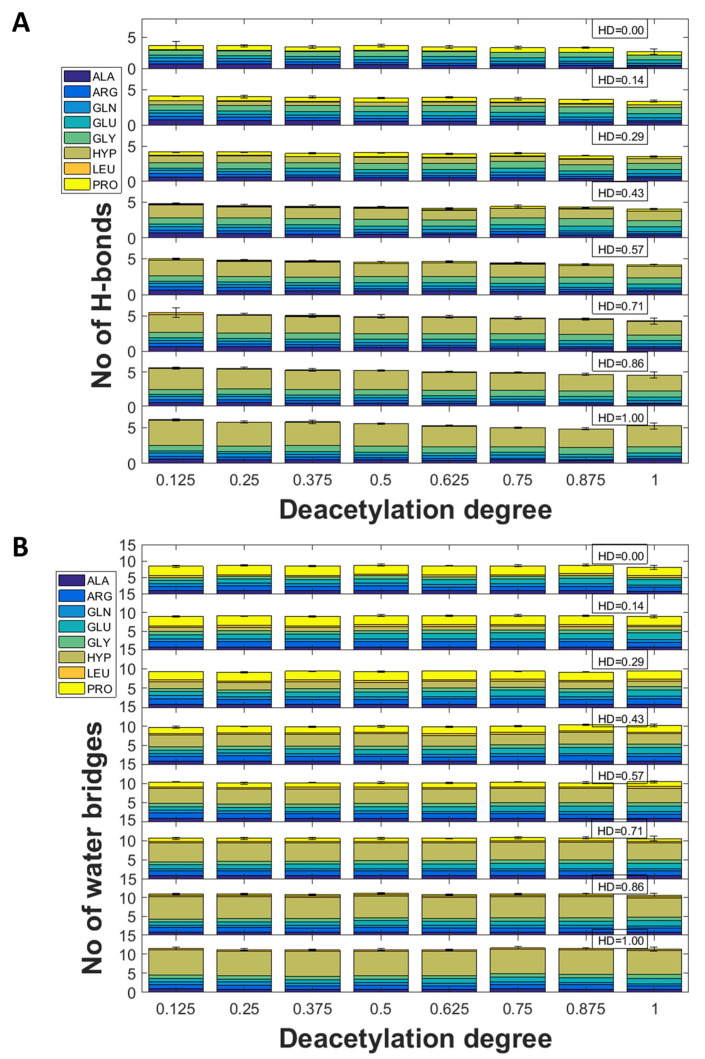
The distributions of direct (**A**) and indirect (**B**) hydrogen bonds identified in different collagen-chitosan complexes.

Interestingly, although the examples presented above suggest a significant influence of the DD on the donor–acceptor character, there are no significant changes in terms of the number of interactions. The distributions of direct and water-mediated H-bonds are presented in Figure 4. As one can see, the number of hydrogen bonds decreases with the increase in DD. 

In the case of water bridges, the number of contacts is not affected by the DD. As one can see, in the case of HD = 0, most of the interactions are formed by PRO. However, the sum of other H-bonds is higher. Surprisingly, when considering both direct and indirect H-bonds, in the case of HD = 1, most of the contacts are formed by HYP. This suggests that the interactions employing the hydroxyl group attached to PRO are more favorable than other H-bonds.

### 2.2. Hydrophobic Interactions

**Figure 5:**
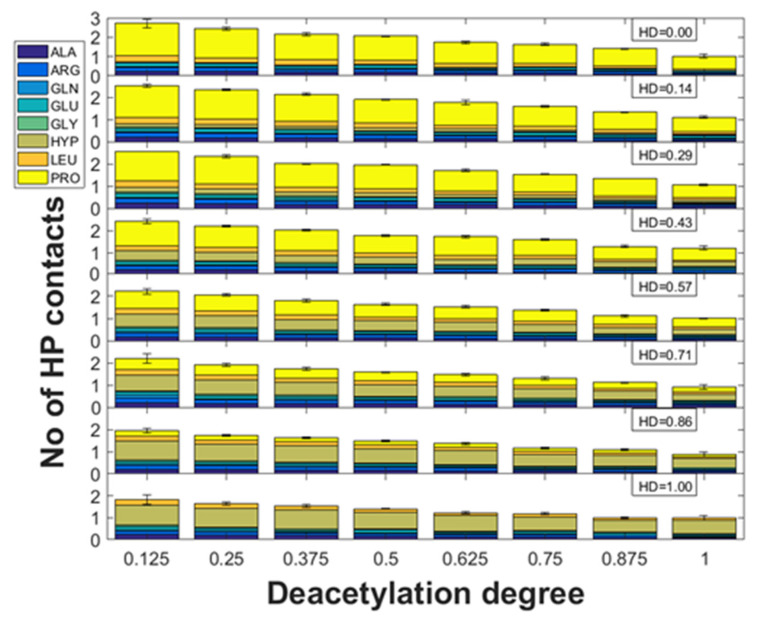
Number of hydrophobic (HP) contacts vs. chitosan DD for different HD of collagen.

As it is documented in Figure 5 the number of hydrophobic (HP) interactions for all different HD states decreases with the increase in DD. Interestingly, most of the contacts of this type are formed by PRO or HYP. This is understandable, since HP forces are formed by the aliphatic groups including CH_3_ in the acetyl moiety. Noteworthy, in the case of all collagen structures, there is a very good correlation between the number of hydrophobic interactions and DD (R^2^ ranges from 0.98 to 0.99). The inspection of the slope values indicates that the complexes employing collagen characterized by HD = 0 are the most sensitive to DD. It was found the most susceptible residue for the decrease in hydrophobic interactions was PRO; however, the slope was strongly related to HD.

### 2.3. Ionic Interactions

**Figure 6:**
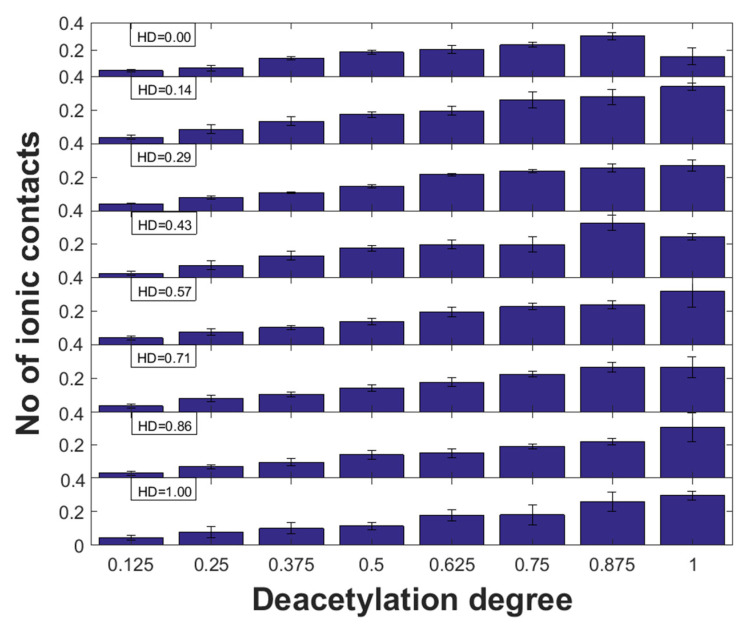
Number of ionic contacts vs. chitosan DD for different HD of collagen.

The influence of DD on the formation of electrostatic interactions is associated with the increase in amino groups which undergo protonation. Noteworthy, the amino nitrogen atom attached to the aliphatic chain is characterized by a much more basic character than the delocalized lone electron pairs in the acetyl group. According to expectation, the ionic interactions are formed by the ionized GLU carboxyl group in the amino acid residue and the ammonium NH_3_^+^ group in chitosan (DD = 1). The contacts between COO^−^ and -NH_3_^+^ were confirmed experimentally for collagen and gelatin blends containing chitosan by using FTIR spectroscopy [27,54,55]. The distributions of these interactions for the complexes characterized by different HD and DD were presented in Figure 6. Noteworthy, in the case of all collagen structures, there is a very good correlation between the number of ionic interactions and DD (R^2^ ranges from 0.6 to 0.97). Since, there was no significant change in slope value between HD, the HYP/PRO ratio generally in most cases does not affect the ionic interactions. However, in the case of HD = 0 and HD = 0.43, a decrease in the number of ionic contacts can be observed for DD = 1. In the case of HD = 0.29 and HD = 0.71, the number of interactions of this type is almost constant for DD = 0.875 and DD = 1. This apparently abnormal behavior is caused by the formation of mainly hydrogen bonds between GLU and hydroxyl groups in chitosan due to conformations that favour positions further from GLU located at the canter of helices.

**Figure 7:**
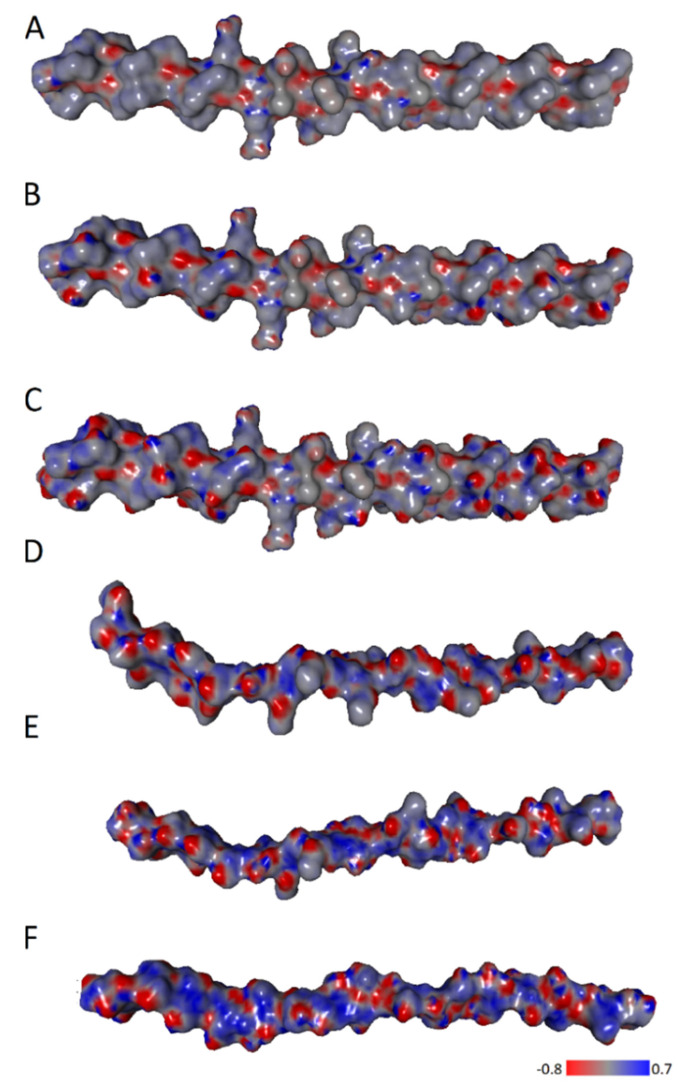
ESP for (**A**) Collagen HD = 0, (**B**) Collagen HD = 0.43, (**C**) Collagen HD = 1, (**D**) chitosan DD = 0.125, (**E**) chitosan DD = 0.5, (**F**) chitosan DD = 1.

Since the ionic forces are directly related to the charge distribution the electrostatic potential maps were determined (Figure 7). The characteristic zwitterionic structure of the peptide necessitates significant variations in charge density. Hence, in the case of collagen and chitosan, many centers with localized positive and negative charges can be noticed.

### 2.4. Entropic and Enthalpic Contributions to Solvation

**Figure 8:**
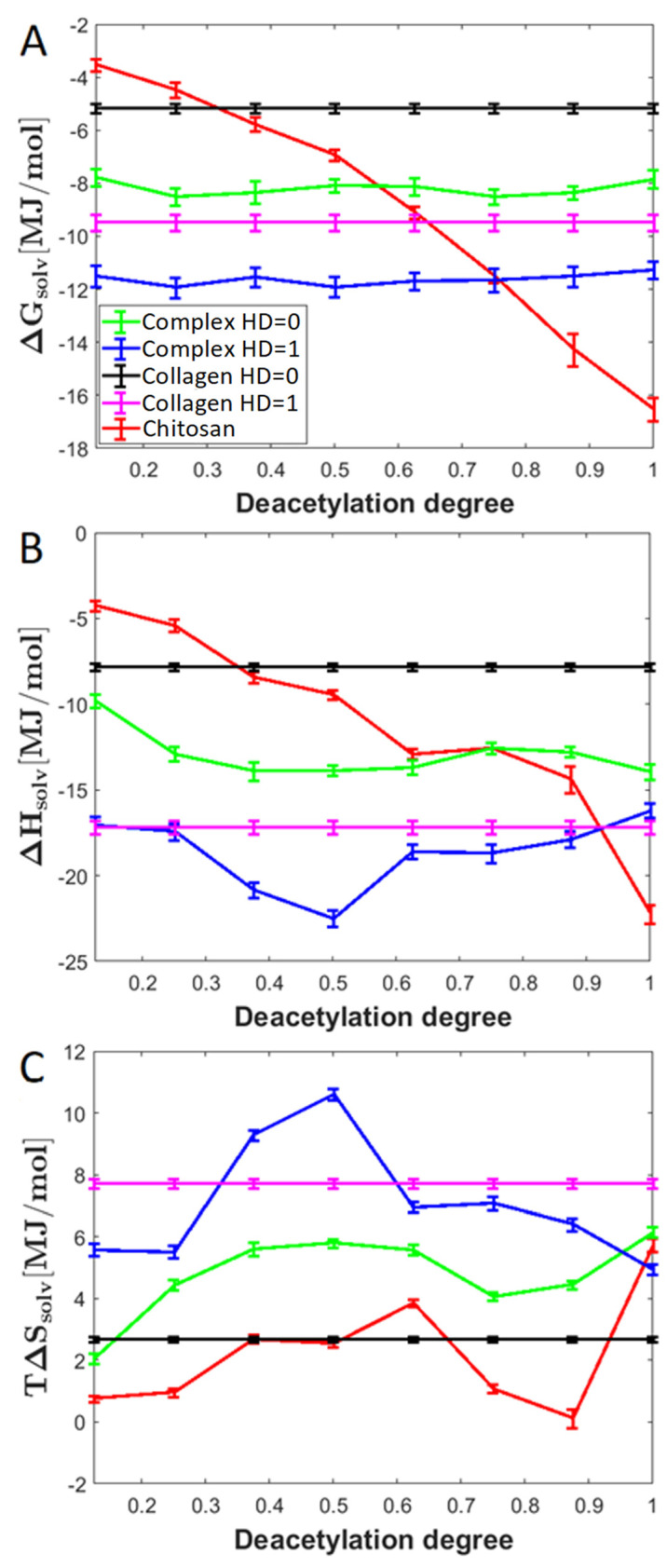
The relationship between solvation Gibbs free energy (ΔG_solv_) and DD (**A**) along with enthalpic (**B**) and entropic (**C**) contributions to solvation for exemplary complexes (HD = 0 and HD = 1) and pure components.

Solvation is a crucial factor affecting the overall complex stability. As expected, the HD affects the solvation efficiency expressed by the free energy of solvation (Figure 8). Interestingly, the enthalpic absolute contributions to the free energy are higher than the entropic one. When considering the chitosan molecule interacting with collagen, DD significantly influences ΔG_solv_. The downhill trend is a consequence of the increase in the number of highly hydrophilic amino groups and the decrease in acetyl moieties. On the other hand, when considering complexes formed by particular collagen analogues, there is a very weak effect of DD on the free energy of solvation. As expected, the solvation of the complexes formed by the completely hydroxylated collagen analogue (HD = 1) is more efficient than in the case of the dehydroxylated collagen. Interestingly, in all cases, the ΔG_solv_ value of the complexes is higher than the sum of ΔG_solv_ values of peptide and chitosan. This indicates that the acidic and basic centers in the considered biopolymers take part in interactions with each other, at the expense of solvation. 

## 3. Methods

### 3.1. Docking and Molecular Dynamics Simulation Setup

In this study, the collagen II structure, (Pro-Hyp-Gly)3-Arg-Ala-Gly-Glu-Pro-Gly-Leu-Gln-Gly-Pro-Ala-Gly-(Pro-Hyp-Gly)3 obtained from the protein data bank (PDB) (code 6JEC 10.2210/pdb6JEC/pdb) was used. In this structure, twelve amino acid residues form a common motif characteristic for human type II collagen. In order to stabilize the triple-helical assembly, the peptide chains were ended with (Pro-Hyp-Gly)3 triplets. In the current study we used trans-4-Hydroxy-L-proline as it is the most common type of HYP present in collagen [56].

The hydroxylation degree in the collagen chain was modified by adding or removing OH groups from PRO and HYP moieties. Peptide structures characterized by the following HD: 0, 6 (HD = 0.14), 12 (HD = 0.29), 18 (HD = 0.43, original structure), 24 (HD = 0.57), 30 (HD = 0.72), 36 (HD = 0.86), and 42 (HD = 1) were taken into account. The positions of HYP/PRO residues for each of ten variants for given HD are provided in Table S1 in Supplementary information. For each HD a set of randomly hydroxylated structures were constructed. Random positions have been generated using the random number generator built in MATLAB [57]. Ten distinct PRO/HYP sequences of available positions for a given HD have been generated based on these random numbers. Randomization of the PRO/HYP position was applied in order to have more statistically significant results. A similar procedure was applied in deacetylation on chitosan, where 5 variations of each DD were prepared to take into account randomization of acetyl group position in chitosan of a given DD. It should be also taken into account that PRO can be hydroxylated in position 3. However, this modification is much more important for collagen IV than collagen II [58]. Furthermore 4-hydroxylation is important from the collagen tertiary structure perspective [32,59,60]. 

The next effect, namely chitosan deacetylation was visualized in Figure 1b. For this purpose, the chitin structure was retrieved from the PubChem database (800 Da) and used to construct chitosan structures characterized by DD = 12.5–100%. 

Finally, all collagen and chitosan structures were subjected to geometry optimization. The docking procedure was performed with an aid of VINA method [61] using default settings. The used for collagen (receptor) initial point charges were determined using AMBER14 force field [62], while in the case of chitosan (ligand) GLYCAM06 force field [63] was applied. Simulation box containing complexes of interest was filled with water and chloride ions to keep the system electrostatically neutral. Noteworthy, the AMBER14 force field applied for collagen shows good recreation of protein dynamics and ensures that the proper configurations and conformations of residues are maintained [64,65,66]. All calculations were carried out using the YASARA software [67]. The most stable complexes of 50 runs were selected by taking into account the 42 kJ/mol free binding energy threshold. As a result, about 1000 complexes for every variant of given hydroxylated collagen and deacetylated chitosan molecule pair. Structures characterized by the native collagen sequence (HD = 0.43), as well as ones with all hydroxylated (HD = 1) and none hydroxylated PRO residues (HD = 0) have only one variant; therefore, those formed only ~150 complexes each. In total, we have obtained ~40,000 complexes that were further used.

In order to include the solvation effect, the molecular dynamics (MD) simulations were performed in water. The applied procedure included the optimization of hydrogen bonds (H-bonds) to achieve appropriate protonation microstates (pH = 7.0) [53]. Then, after achieving the steepest gradient during the annealing simulations, the MD calculations were carried out for one ns using the three different forcefields namely, AMBER14 for collagen, GLYCAM06 for chitosan, and TIP3P for water. The default AMBER settings, namely 10 Å-threshold for van der Waals interactions, and no threshold was applied for Particle Mesh Ewald algorithm [68], used for the electrostatic forces identification. The following default settings [67] were used for motions equations integration: 1.25 fs multiple time steps in case of bonded interactions and 2.5 fs for non-bonded ones, T = 310 K, P = 1 atm, NPT ensemble, protocol. After the RMSD (root mean square deviation/displacement) analysis of MD simulations, an arithmetic average of 100 points from the 0.9–1 ns range (0.01 ns sampling) were selected for determination of free energy of binding. The analogical procedure was applied for the numbers of intermolecular interactions.

In order to evaluate the structural consequences of DD and HD expressed by the alternations in the distributions of particular types of interactions (direct and water-mediated hydrogen bonds, ionic and hydrophobic forces), the default YASARA [67] definitions were applied for mentioned contacts. 

### 3.2. Binding Free Energy Determination

In this study, the binding free energy was calculated by using a single trajectory approach (Molecular Mechanics/Poisson–Boltzmann Surface Area (MM/PBSA) and the YASARA Structure). For this purpose, the APBS (Adaptive Poisson–Boltzmann Solver) and AMBER14 force field was utilized for solvation energy determination and include electrostatics [69,70]. The binding free energy, ∆G_b_, was determined for optimized structures using the Equation (2).

(2) $$\Delta G_{b}=\Delta G_{c}\;–\left(\Delta G_{L}+\Delta G_{R}\right)$$

where ∆G_*c*_, ∆G_*L*_, and ∆G_*R*_ denote the free energy of the chitosan–collagen complex, the ligand, which is chitosan and the receptor (collagen type II). 

### 3.3. Determination of Free Energy and Entropy of Solvation

The free energy and entropy of solvation were determined based on the approach presented in the study of Syme et al. [71]. The free energy of solvation (ΔG_solv_) was calculated by subtracting the free energy for the unsolvated form from the free energy value corresponding to the solvated form. On the other hand, the entropy of solvation at a given temperature (ΔG_T_) can be calculated from the following equation:

(3) $$\Delta S_{solv}\left(T\right)=-\frac{\left[\Delta G_{solv}\left(T+\Delta T\right)-\Delta G_{solv}\left(T-\Delta T\right)\right]}{2\Delta T}$$

where Δ*T* denotes temperature difference, for which the additional MD simulations were performed. The value of entropy is an average from 70 structures for two types of collagen analogues characterized by the highest and the lowest HYP/PRO ratio (HD = 0 and HD = 1).

## 4. Conclusions

Collagen–chitosan blends are very often explored biomaterials due to their unique properties. In this study, the effect of collagen PRO residues hydroxylation and chitosan deacetylation was analyzed using MD methodology. Both HD and DD are crucial factors, which are known to affect the important properties of considered biopolymers. It could be observed that for all collagen type II analogues a linear relationship between the Gibbs binding free energy and DD ratio exists. Apart from the energetical features, the important structural parameters such as the number of H-bonds, hydrophobic interactions and ionic contacts were determined. It can be concluded that the high affinity of collagen to completely deacetylated chitosan is caused by a large number of amino groups in chitosan which increases the basic character. 

The obtained results are quite intuitive and consistent with some experimental studies reported in the literature. The used collagen structures represent a fragment of human collagen type II characterized by the tertiary structure arrangement and the presence of the crucial residues and fragments (particularly the most common triplet PRO-HYP-GLY). Although, the applied peptide helices are relatively short they seem to be sufficient to show how the HYP/PRO ratio can influence the chitosan affinity for collagen. However, there are some aspects which are worth considering in further studies, such as the influence of the number of individual residues on the collagen affinity for chitosan. This seems to be important in the context of the polyelectrolytic nature of collagen. Therefore, the obtained results, are a good starting point for examining more complex systems. For this purpose a coarse-grained model seems to be a promising tool. In addition, the results presented in this study indicate the important factors affecting collagen-chitosan stability that are worth investigating experimentally.

## Data Availability

All data (MD simulation details) are available in the manuscript or upon request to the corresponding author.

## Supplementary Materials

The following supporting information can be downloaded at: https://www.mdpi.com/article/10.3390/molecules28010154/s1, Figure S1: The oxygen atoms numbering in units of chitin.; Table S1: The HYP positions presented on the collagen structures used in the study; Table S2: Distribution of H-bonds between chitosan and collagen amino acid atoms for HD = 0 and DD = 1; Table S3: Distribution of H-bonds between chitosan and collagen amino acid atoms for HD = 0.42 and DD = 1; Table S4: Distribution of H-bonds between chitosan and collagen amino acid atoms for HD = 1 and DD = 1; Table S5: Distribution of H-bonds between chitosan and collagen amino acid atoms for HD = 0 and DD = 0.125; Table S6: Distribution of H-bonds between chitosan and collagen amino acid atoms for HD = 0.42 and DD = 0.5; Table S7: Distribution of H-bonds between chitosan and collagen amino acid atoms for HD = 1 and DD = 0.125; Table S8: H-bonds lengths [Å] between chitosan and collagen amino acid atoms for HD = 0 and DD = 1; Table S9: H-bonds lengths [Å] between chitosan and collagen amino acid atoms for HD = 0.42 and DD = 1; Table S10: H-bonds lengths [Å] between chitosan and collagen amino acid atoms for HD = 1 and DD = 1; Table S11: H-bonds lengths [Å] between chitosan and collagen amino acid atoms for HD = 0 and DD = 0.125; Table S12: H-bonds lengths [Å] between chitosan and collagen amino acid atoms for HD = 0.42 and DD = 0.5; Table S13: H-bonds lengths [Å] between chitosan and collagen amino acid atoms for HD = 1 and DD = 0.125; Table S14: Energies of H-bonds [kJ/mol] between chitosan and given collagen amino acid residue atom for HD = 0 and DD = 1; Table S15: Energies of H-bonds [kJ/mol] between chitosan and given collagen amino acid residue atom for HD = 0.42 and DD = 1; Table S16: Energies of H-bonds [kJ/mol] between chitosan and given collagen amino acid residue atom for HD = 1 and DD = 1; Table S17: Energies of H-bonds [kJ/mol] between chitosan and given collagen amino acid residue atom for HD = 0 and DD = 0.125; Table S18: Energies of H-bonds [kJ/mol] between chitosan and given collagen amino acid residue atom for HD = 0.42 and DD = 0.5; Table S19: Energies of H-bonds [kJ/mol] between chitosan and given collagen amino acid residue atom for f HD = 1 and DD = 0.125; Table S20: The H-bonds acceptor-donor frequency analysis. Values in cells represent how often chitosan in a given interaction plays the role of acceptor for HD = 0 and DD = 1; Table S21 The H-bonds acceptor-donor frequency analysis. Values in cells represent how often chitosan in a given interaction plays the role of acceptor for HD = 0.42 and DD = 1; Table S22: The H-bonds acceptor-donor frequency analysis. Values in cells represent how often chitosan in a given interaction plays the role of acceptor for HD = 1 and DD = 1; Table S23 The H-bonds acceptor-donor frequency analysis. Values in cells represent how often chitosan in a given interaction plays the role of acceptor for HD = 0 and DD = 0.125; Table S24 The H-bonds acceptor-donor frequency analysis. Values in cells represent how often chitosan in a given interaction plays the role of acceptor for HD = 0.42 and DD = 0.5; Table S25: The H-bonds acceptor-donor frequency analysis. Values in cells represent how often chitosan in a given interaction plays the role of acceptor for HD = 1 and DD = 0.125.
