# Supplementary material for: Collagen Type II—Chitosan Interactions as Dependent on Hydroxylation and Acetylation Inferred from Molecular Dynamics Simulations
Source: Molecules. 2022 Dec 24;28(1):154. doi: 10.3390/molecules28010154 (PMC9821911; doi:10.3390/molecules28010154)
Supplement: Supplementary file 1 [file molecules-28-00154-s001.zip › Supplementary materials.pdf]

Supplementary Information

Collagen Type II – Chitosan interactions as dependent on hydroxylation and acetylation inferred from molecular dynamics simulations

Maciej Przybyłek [1]*, Piotr Bełdowski [2,3], D.C. Florian Wieland[3]
Piotr Cysewski [1] , Alina Sionkowska [4]*

[1] Department of Physical Chemistry, Pharmacy Faculty, Collegium Medicum of Bydgoszcz, Nicolaus Coper-nicus University in Toruń, Kurpińskiego 5, 85-950 Bydgoszcz, Poland

[2] Institute of Mathematics and Physics, Bydgoszcz University of Science and Technology, al. Kaliskiego 7, 85-796 Bydgoszcz, Poland.

[3] Helmholtz-Zentrum hereon: Institute for metallic Biomaterials, Max-Planck-Straße 1, 21502 Geesthacht, Germany

[4] Department of Biomaterials and Cosmetics Chemistry, Faculty of Chemistry, Nicolaus Copernicus University in Toruń, Gagarin 7, 87-100 Toruń, Poland

Table of Contents

Figure S1. The oxygen atoms numbering in units of chitin. 3

Table S1. The HYP positions presented on the collagen structures used in the study. The first letter links to the chain and the second a position in the primary structure. 4

Table S2 Distribution of H-bonds between chitosan and collagen amino acid atoms for HD=0 and DD=1. Percentages represent the contributions of H-bonds identified between chitosan and a given amino acid residue. 7

Table S3 Distribution of H-bonds between chitosan and collagen amino acid atoms for HD=0.42 and DD=1. Percentages represent the contributions of H-bonds identified between chitosan and a given amino acid residue. The numbering of atoms is the same as in Table. S1 and Figure S1. 7

Table S4 Distribution of H-bonds between chitosan and collagen amino acid atoms for HD=1 and DD=1. Percentages represent the contributions of H-bonds identified between chitosan and a given amino acid residue. The numbering of atoms is the same as in Table. S1 and Figure S1. 8

Table S5 Distribution of H-bonds between chitosan and collagen amino acid atoms for HD=0 and DD=0.125. Percentages represent the contributions of H-bonds identified between chitosan and a given amino acid residue. The numbering of atoms is the same as in Table. S1 and Figure S1. 8

Table S6 Distribution of H-bonds between chitosan and collagen amino acid atoms for HD=0.42 and DD=0.5. Percentages represent the contributions of H-bonds identified between chitosan and a given amino acid residue. The numbering of atoms is the same as in Table. S1 and Figure S1. 8

Table S7 Distribution of H-bonds between chitosan and collagen amino acid atoms for HD=1 and DD=0.125. Percentages represent the contributions of H-bonds identified between chitosan and a given amino acid residue. The numbering of atoms is the same as in Table. S1 and Figure S1. 9

Table S8 H-bonds lengths [Å] between chitosan and collagen amino acid atoms for HD=0 and DD=1. The numbering of atoms is the same as in Table. S1 and Figure S1. 9

Table S9 H-bonds lengths [Å] between chitosan and collagen amino acid atoms for HD=0.42 and DD=1. The numbering of atoms is the same as in Table. S1 and Figure S1. 10

Table S10 H-bonds lengths [Å] between chitosan and collagen amino acid atoms for HD=1 and DD=1. The numbering of atoms is the same as in Table. S1 and Figure S1.. 10

Table S11 H-bonds lengths [Å] between chitosan and collagen amino acid atoms for HD=0 and DD=0.125. The numbering of atoms is the same as in Table. S1 and Figure S1................................... 10

Table S12 H-bonds lengths [Å] between chitosan and collagen amino acid atoms for HD=0.42 and DD=0.5. The numbering of atoms is the same as in Table. S1 and Figure S1....................................... 11

Table S13 H-bonds lengths [Å] between chitosan and collagen amino acid atoms for HD=1 and DD=0.125. The numbering of atoms is the same as in Table. S1 and Figure S1................................... 11

Table S20 The H-bonds acceptor-donor frequency analysis. Values in cells represent how often chitosan in a given interaction plays the role of acceptor for HD=0 and DD=1. 14

Table S21 The H-bonds acceptor-donor frequency analysis. Values in cells represent how often chitosan in a given interaction plays the role of acceptor for HD=0.42 and DD=1. 14

Table S22 The H-bonds acceptor-donor frequency analysis. Values in cells represent how often chitosan in a given interaction plays the role of acceptor for HD=1 and DD=1. 15

Table S23 The H-bonds acceptor-donor frequency analysis. Values in cells represent how often chitosan in a given interaction plays the role of acceptor for HD=0 and DD=0.125. 15

Table S24 The H-bonds acceptor-donor frequency analysis. Values in cells represent how often chitosan in a given interaction plays the role of acceptor for HD=0.42 and DD=0.5. 16

Table S25 The H-bonds acceptor-donor frequency analysis. Values in cells represent how often chitosan in a given interaction plays the role of acceptor for HD=1 and DD=0.125. 16

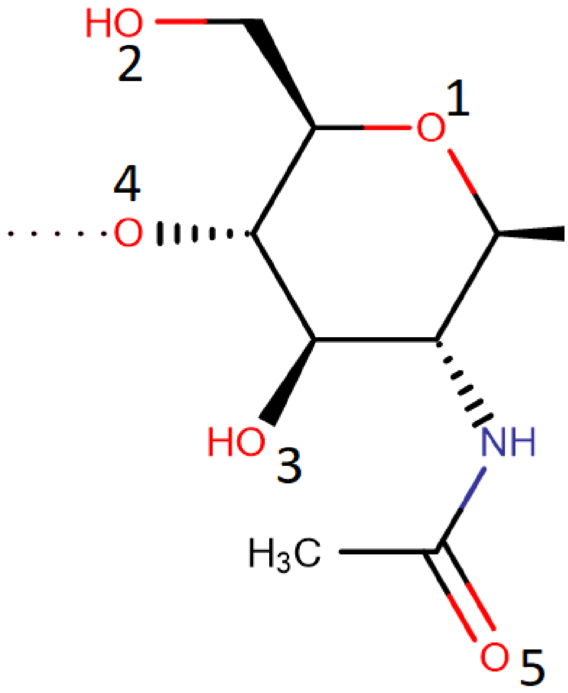

Figure S1. The oxygen atoms numbering in units of chitin.

Table S1. The HYP positions presented on the collagen structures used in the study. The first letter links to the chain and the second a position in the primary structure.

Hydroxylation degree	HYP position in the sequence
**0.00**	No HYP residues, only PRO residues present
**0.14**	B8-C2-C5-C8-C23-C29<br>A2-B5-B29-C2-C8-C26<br>A5-A23-A26-B2-B5-C8<br>A2-A8-A29-B8-C2-C29<br>A2-A5-A23-B2-B5-B8<br>A8-B2-B5-B29-C2-C5<br>A2-A8-B23-C5-C23-C29<br>A2-A5-A8-A23-C26-C29<br>A26-A29-B2-B29-C2-C5<br>A2-A5-B2-B23-B29-C29
**0.29**	A2-A5-A23-A29-B2-B5-B23-B26-C2-C8-C23-C29<br>A5-A8-A23-A26-A29-B5-B23-C2-C5-C8-C23-C29<br>A5-A23-A26-A29-B2-B23-B29-C2-C5-C23-C26-C29<br>A2-A8-A29-B2-B23-B26-B29-C5-C8-C23-C26-C29<br>A2-A5-A8-A23-A26-A29-B2-B8-B23-B26-B29-C26<br>A2-A5-A23-B5-B8-B23-B26-C5-C8-C23-C26-C29<br>A5-A8-A23-A26-A29-B2-B5-B8-B23-B26-B29-C8<br>A2-A5-A23-A26-A29-B8-B23-B26-B29-C8-C26-C29<br>A2-A5-A23-A26-A29-B2-B26-B29-C2-C5-C8-C29<br>A2-A5-A8-A23-A26-A29-B2-B5-B8-C2-C8-C23
**0.43**	HYP-A2-A5-A8-A23-A26-A29-B2-B5-B8-B23-B26-B29-C2-C5-C8-C23-C26-C29<br>PRO-A1-A4-A7-A14-A19-A22-A25-A28-B1-B4-B7-B14-B19-B22-B25-B28-C1-C4-C7-C14-C19-C22-C25-C28

Hydroxylation degree	HYP position in the sequence
**0.57**	A2-A5-A7-A8-A23-A26-A29-B1-B2-B4-B5-B7-B8-B23-B26-B28-B29-C2-C5-C8-C14-C23-C26-C29<br>A2-A5-A8-A22-A23-A26-A29-B2-B4-B5-B8-B22-B23-B26-B28-B29-C2-C5-C8-C14-C23-C26-C28-C29<br>A2-A5-A8-A19-A22-A23-A26-A29-B1-B2-B5-B7-B8-B23-B26-B29-C2-C4-C5-C8-C22-C23-C26-C29<br>A2-A5-A8-A23-A26-A29-B2-B5-B8-B14-B19-B23-B25-B26-B29-C2-C5-C8-C14-C22-C23-C25-C26-C29<br>A2-A4-A5-A7-A8-A22-A23-A26-A29-B2-B5-B8-B23-B26-B29-C2-C5-C7-C8-C23-C25-C26-C28-C29<br>A2-A5-A7-A8-A19-A23-A25-A26-A28-A29-B2-B5-B7-B8-B23-B26-B29-C2-C5-C8-C19-C23-C26-C29<br>A2-A5-A7-A8-A14-A19-A23-A26-A29-B1-B2-B4-B5-B8-B23-B26-B28-B29-C2-C5-C8-C23-C26-C29<br>A1-A2-A5-A7-A8-A23-A25-A26-A29-B2-B5-B8-B23-B25-B26-B28-B29-C2-C5-C8-C14-C23-C26-C29<br>A2-A5-A8-A14-A19-A22-A23-A26-A29-B2-B5-B8-B22-B23-B26-B29-C2-C5-C8-C19-C23-C26-C28-C29<br>A2-A5-A8-A19-A23-A25-A26-A28-A29-B1-B2-B5-B8-B19-B22-B23-B26-B29-C2-C5-C8-C23-C26-C29
**0.71**	A1-A2-A4-A5-A8-A14-A22-A23-A26-A28-A29-B2-B4-B5-B7-B8-B22-B23-B26-B28-B29-C2-C4-C5-C8-C23-C25-C26-C28-C29<br>A2-A5-A7-A8-A14-A22-A23-A25-A26-A28-A29-B2-B5-B7-B8-B22-B23-B25-B26-B29-C1-C2-C5-C7-C8-C14-C22-C23-C26-C29<br>A1-A2-A4-A5-A8-A19-A22-A23-A26-A29-B2-B4-B5-B8-B14-B19-B23-B26-B29-C1-C2-C5-C7-C8-C19-C22-C23-C25-C26-C29<br>A2-A4-A5-A7-A8-A14-A22-A23-A25-A26-A28-A29-B1-B2-B5-B8-B22-B23-B26-B29-C1-C2-C4-C5-C8-C14-C23-C26-C28-C29<br>A1-A2-A5-A8-A14-A22-A23-A26-A29-B1-B2-B4-B5-B8-B14-B19-B23-B26-B28-B29-C2-C4-C5-C7-C8-C14-C22-C23-C26-C29<br>A1-A2-A4-A5-A8-A14-A19-A23-A25-A26-A29-B1-B2-B5-B8-B14-B22-B23-B25-B26-B29-C1-C2-C5-C8-C14-C23-C26-C28-C29<br>A2-A4-A5-A7-A8-A19-A22-A23-A26-A29-B1-B2-B5-B7-B8-B14-B19-B23-B25-B26-B28-B29-C1-C2-C5-C8-C22-C23-C26-C29<br>A2-A4-A5-A7-A8-A19-A22-A23-A26-A29-B1-B2-B4-B5-B8-B23-B25-B26-B29-C1-C2-C4-C5-C7-C8-C19-C22-C23-C26-C29<br>A2-A4-A5-A7-A8-A14-A22-A23-A25-A26-A28-A29-B2-B5-B7-B8-B14-B19-B23-B26-B29-C2-C5-C8-C19-C22-C23-C25-C26-C29<br>A1-A2-A5-A8-A14-A19-A22-A23-A25-A26-A29-B2-B5-B7-B8-B22-B23-B26-B29-C2-C5-C8-C14-C19-C22-C23-C25-C26-C28-C29

Hydroxylation degree	HYP position in the sequence
**0.86**	A1-A2-A4-A5-A8-A14-A22-A23-A26-A29-B2-B4-B5-B7-B8-B14-B19-B22-B23-B25-B26-B28-B29-C1-C2-C4-C5-C7-C8-C14-C19-C22-C23-C25-C26-C29<br>A1-A2-A4-A5-A7-A8-A14-A19-A22-A23-A26-A28-A29-B1-B2-B4-B5-B7-B8-B22-B23-B25-B26-B28-B29-C2-C4-C5-C7-C8-C19-C22-C23-C25-C26-C29<br>A1-A2-A5-A7-A8-A14-A19-A22-A23-A25-A26-A29-B1-B2-B4-B5-B7-B8-B14-B19-B23-B26-B28-B29-C2-C4-C5-C7-C8-C19-C22-C23-C25-C26-C28-C29<br>A1-A2-A4-A5-A7-A8-A14-A19-A22-A23-A25-A26-A28-A29-B2-B4-B5-B8-B14-B22-B23-B26-B28-B29-C1-C2-C5-C7-C8-C19-C22-C23-C25-C26-C28-C29<br>A2-A4-A5-A7-A8-A14-A22-A23-A25-A26-A28-A29-B1-B2-B5-B7-B8-B14-B23-B26-B28-B29-C1-C2-C4-C5-C7-C8-C14-C19-C22-C23-C25-C26-C28-C29<br>A2-A4-A5-A7-A8-A19-A23-A25-A26-A28-A29-B1-B2-B4-B5-B7-B8-B14-B19-B23-B25-B26-B28-B29-C1-C2-C4-C5-C7-C8-C14-C19-C23-C26-C28-C29<br>A1-A2-A5-A7-A8-A14-A19-A23-A25-A26-A29-B1-B2-B5-B7-B8-B14-B19-B23-B25-B26-B28-B29-C1-C2-C4-C5-C7-C8-C14-C19-C22-C23-C25-C26-C29<br>A2-A4-A5-A7-A8-A14-A19-A22-A23-A25-A26-A28-A29-B1-B2-B5-B8-B14-B19-B22-B23-B25-B26-B29-C1-C2-C4-C5-C8-C14-C22-C23-C25-C26-C28-C29<br>A2-A4-A5-A7-A8-A19-A22-A23-A25-A26-A28-A29-B1-B2-B4-B5-B8-B14-B19-B23-B25-B26-B28-B29-C2-C4-C5-C7-C8-C14-C22-C23-C25-C26-C28-C29<br>A1-A2-A4-A5-A8-A14-A22-A23-A25-A26-A28-A29-B1-B2-B5-B7-B8-B14-B19-B22-B23-B25-B26-B28-B29-C1-C2-C5-C7-C8-C19-C23-C25-C26-C28-C29
**1.00**	No PRO residues, only HYP residues present

Table S2 Distribution of H-bonds between chitosan and collagen amino acid atoms for HD=0 and DD=1. Percentages represent the contributions of H-bonds identified between chitosan and a given amino acid residue.

	ARG	ARG	ARG	ARG	GLU	GLU	GLU	HYP	HYP	PRO	PRO	GLY	ALA	ALA	GLN	GLN	GLN	GLN
	**N**	**NH1**	**NH2**	**NE**	**OE1**	**OE2**	**N**	**OD1**	**O**	**N**	**O**	**O**	**N**	**O**	**OE1**	**NE2**	**O**	**N**
**N**	0%	33%	0%	17%	7%	10%	0%	0%	0%	0%	44%	30%	7%	20%	13%	0%	13%	0%
**O1**	0%	0%	0%	0%	0%	0%	0%	0%	0%	0%	0%	0%	0%	0%	0%	0%	0%	0%
**O2**	0%	50%	0%	0%	28%	10%	7%	0%	0%	0%	26%	37%	7%	40%	20%	7%	7%	7%
**O3**	0%	0%	0%	0%	14%	17%	0%	0%	0%	0%	26%	26%	20%	7%	7%	13%	7%	7%
**O4**	0%	0%	0%	0%	7%	0%	0%	0%	0%	0%	4%	7%	0%	0%	0%	0%	0%	0%
**O5**	0%	0%	0%	0%	0%	0%	0%	0%	0%	0%	0%	0%	0%	0%	0%	0%	0%	0%

ARG - NE, HH1 and NH2 guanidino group of the side chain, N - nitrogen of the amino group in the main chain, GLU - OE1 and OE2 oxygen atoms from the carboxyl group, N - nitrogen of the amino group in the main chain; HYP - OD1 - oxygen from the hydroxyl group, N - nitrogen of the amino group in the main chain; PRO - N - nitrogen of the amino group in the main chain, O - oxygen in the main chain; GLY - N - nitrogen of the amino group in the main chain, O - oxygen in the main chain; ALA - N - nitrogen of the amino group in the main chain, O - oxygen in the main chain; The numbering of atoms in Chitosan according to Fig. S1

Table S3 Distribution of H-bonds between chitosan and collagen amino acid atoms for HD=0.42 and DD=1. Percentages represent the contributions of H-bonds identified between chitosan and a given amino acid residue. The numbering of atoms is the same as in Table. S1 and Figure S1.

	ARG	ARG	ARG	ARG	GLU	GLU	GLU	HYP	HYP	PRO	PRO	GLY	ALA	ALA	GLN	GLN	GLN	GLN
	**N**	**NH1**	**NH2**	**NE**	**OE1**	**OE2**	**N**	**OD1**	**O**	**N**	**O**	**O**	**N**	**O**	**OE1**	**NE2**	**O**	**N**
**N**	17%	0%	0%	0%	19%	8%	3%	19%	16%	0%	42%	33%	0%	44%	21%	0%	5%	5%
**O1**	0%	0%	0%	17%	0%	0%	0%	2%	0%	0%	0%	0%	6%	0%	0%	0%	0%	0%
**O2**	17%	17%	17%	0%	6%	19%	3%	13%	11%	8%	17%	44%	17%	22%	5%	5%	0%	26%
**O3**	0%	17%	0%	0%	17%	22%	0%	16%	21%	0%	33%	23%	0%	11%	11%	16%	0%	0%
**O4**	0%	0%	0%	0%	3%	0%	0%	2%	2%	0%	0%	0%	0%	0%	0%	5%	0%	0%
**O5**	0%	0%	0%	0%	0%	0%	0%	0%	0%	0%	0%	0%	0%	0%	0%	0%	0%	0%

Table S4 Distribution of H-bonds between chitosan and collagen amino acid atoms for HD=1 and DD=1. Percentages represent the contributions of H-bonds identified between chitosan and a given amino acid residue. The numbering of atoms is the same as in Table. S1 and Figure S1.

	ARG	ARG	ARG	ARG	GLU	GLU	GLU	HYP	HYP	PRO	PRO	GLY	ALA	ALA	GLN	GLN	GLN	GLN
	**N**	**NH1**	**NH2**	**NE**	**OE1**	**OE2**	**N**	**OD1**	**O**	**N**	**O**	**O**	**N**	**O**	**OE1**	**NE2**	**O**	**N**
**N**	9%	9%	0%	0%	14%	7%	4%	28%	13%	0%	0%	34%	6%	19%	21%	5%	11%	0%
**O1**	0%	0%	0%	0%	0%	0%	0%	0%	0%	0%	0%	0%	0%	0%	0%	5%	0%	0%
**O2**	9%	9%	0%	9%	11%	29%	4%	21%	12%	0%	0%	38%	25%	25%	0%	0%	21%	21%
**O3**	9%	9%	18%	18%	29%	4%	0%	14%	8%	0%	0%	28%	19%	6%	16%	0%	0%	0%
**O4**	0%	0%	0%	0%	0%	0%	0%	2%	1%	0%	0%	0%	0%	0%	0%	0%	0%	0%
**O5**	0%	0%	0%	0%	0%	0%	0%	0%	0%	0%	0%	0%	0%	0%	0%	0%	0%	0%

Table S5 Distribution of H-bonds between chitosan and collagen amino acid atoms for HD=0 and DD=0.125. Percentages represent the contributions of H-bonds identified between chitosan and a given amino acid residue. The numbering of atoms is the same as in Table. S1 and Figure S1.

	ARG	ARG	ARG	ARG	GLU	GLU	GLU	HYP	HYP	PRO	PRO	GLY	ALA	ALA	GLN	GLN	GLN	GLN
	**N**	**NH1**	**NH2**	**NE**	**OE1**	**OE2**	**N**	**OD1**	**O**	**N**	**O**	**O**	**N**	**O**	**OE1**	**NE2**	**O**	**N**
**N**	0%	0%	0%	0%	0%	6%	0%	0%	0%	0%	10%	7%	0%	0%	10%	0%	0%	0%
**O1**	0%	8%	4%	0%	0%	0%	0%	0%	0%	0%	0%	0%	3%	0%	0%	0%	0%	0%
**O2**	13%	0%	8%	8%	28%	11%	6%	0%	0%	7%	53%	76%	24%	32%	13%	7%	17%	10%
**O3**	0%	8%	13%	4%	0%	17%	6%	0%	0%	7%	20%	14%	9%	9%	3%	13%	3%	3%
**O4**	0%	4%	0%	0%	11%	0%	0%	0%	0%	0%	0%	3%	0%	3%	0%	0%	0%	0%
**O5**	4%	25%	0%	0%	0%	0%	17%	0%	0%	3%	0%	0%	21%	0%	0%	17%	0%	3%

Table S6 Distribution of H-bonds between chitosan and collagen amino acid atoms for HD=0.42 and DD=0.5. Percentages represent the contributions of H-bonds identified between chitosan and a given amino acid residue. The numbering of atoms is the same as in Table. S1 and Figure S1.

	ARG	ARG	ARG	ARG	GLU	GLU	GLU	HYP	HYP	PRO	PRO	GLY	ALA	ALA	GLN	GLN	GLN	GLN
	**N**	**NH1**	**NH2**	**NE**	**OE1**	**OE2**	**N**	**OD1**	**O**	**N**	**O**	**O**	**N**	**O**	**OE1**	**NE2**	**O**	**N**
**N**	0%	0%	0%	0%	12%	9%	0%	9%	15%	0%	25%	36%	0%	21%	15%	0%	8%	0%
**O1**	0%	5%	0%	0%	0%	0%	0%	3%	0%	0%	0%	0%	5%	0%	0%	0%	0%	0%
**O2**	15%	30%	10%	0%	30%	0%	3%	14%	18%	13%	25%	31%	42%	21%	8%	8%	8%	31%

	ARG	ARG	ARG	ARG	GLU	GLU	GLU	HYP	HYP	PRO	PRO	GLY	ALA	ALA	GLN	GLN	GLN	GLN
	**N**	**NH1**	**NH2**	**NE**	**OE1**	**OE2**	**N**	**OD1**	**O**	**N**	**O**	**O**	**N**	**O**	**OE1**	**NE2**	**O**	**N**
**O3**	0%	5%	5%	0%	21%	12%	3%	19%	8%	13%	13%	31%	11%	0%	0%	0%	8%	8%
**O4**	0%	0%	0%	0%	3%	3%	0%	4%	3%	0%	13%	3%	0%	0%	0%	8%	0%	0%
**O5**	0%	15%	10%	5%	0%	0%	3%	9%	0%	0%	0%	0%	0%	0%	0%	0%	0%	0%

Table S7 Distribution of H-bonds between chitosan and collagen amino acid atoms for HD=1 and DD=0.125. Percentages represent the contributions of H-bonds identified between chitosan and a given amino acid residue. The numbering of atoms is the same as in Table. S1 and Figure S1.

	ARG	ARG	ARG	ARG	GLU	GLU	GLU	HYP	HYP	PRO	PRO	GLY	ALA	ALA	GLN	GLN	GLN	GLN
	**N**	**NH1**	**NH2**	**NE**	**OE1**	**OE2**	**N**	**OD1**	**O**	**N**	**O**	**O**	**N**	**O**	**OE1**	**NE2**	**O**	**N**
**N**	0%	0%	0%	0%	7%	7%	0%	9%	1%	0%	0%	9%	0%	5%	5%	0%	5%	0%
**O1**	0%	0%	0%	0%	0%	0%	0%	2%	0%	0%	0%	0%	0%	0%	0%	0%	0%	0%
**O2**	0%	9%	18%	9%	7%	20%	7%	24%	14%	0%	0%	75%	45%	23%	5%	15%	10%	25%
**O3**	0%	27%	0%	0%	7%	33%	0%	20%	7%	0%	0%	13%	14%	5%	5%	0%	5%	10%
**O4**	0%	9%	0%	0%	7%	0%	0%	3%	1%	0%	0%	3%	0%	0%	0%	0%	0%	0%
**O5**	0%	18%	0%	9%	0%	0%	7%	17%	0%	0%	0%	0%	9%	0%	0%	5%	0%	10%

Table S8 H-bonds lengths [Å] between chitosan and collagen amino acid atoms for **HD=0 and DD=1**. The numbering of atoms is the same as in Table. S1 and Figure S1.

	ARG	ARG	ARG	ARG	GLU	GLU	GLU	HYP	HYP	PRO	PRO	GLY	ALA	ALA	GLN	GLN	GLN	GLN
	**N**	**NH1**	**NH2**	**NE**	**OE1**	**OE2**	**N**	**OD1**	**O**	**N**	**O**	**O**	**N**	**O**	**OE1**	**NE2**	**O**	**N**
**N**	-	1,89	-	1,95	1,87	1,94	-	-	-	-	2,00	2,01	1,87	1,93	2,105	-	1,91	-
**O1**	-	-	-	-	-	-	-	-	-	-	-	-	-	-	-	-	-	-
**O2**	-	2,06	-	-	1,70	1,75	2,04	-	-	-	1,84	1,78	1,98	1,74	1,80	2,19	2,1	2,04
**O3**	-	-	-	-	1,68	1,81	-	-	-	-	1,86	1,86	2,12	1,54	1,65	2,09	1,86	2,32
**O4**	-	-	-	-	1,84	-	-	-	-	-	1,69	1,64	-	-	-	-	-	-
**O5**	-	-	-	-	-	-	-	-	-	-	-	-	-	-	-	-	-	-

Table S9 H-bonds lengths [Å] between chitosan and collagen amino acid atoms for **HD=0.42 and DD=1**. The numbering of atoms is the same as in Table. S1 and Figure S1.

	**ARG**	**ARG**	**ARG**	**ARG**	**GLU**	**GLU**	**GLU**	**HYP**	**HYP**	**PRO**	**PRO**	**GLY**	**ALA**	**ALA**	**GLN**	**GLN**	**GLN**	**GLN**
	**N**	**NH1**	**NH2**	**NE**	**OE1**	**OE2**	**N**	**OD1**	**O**	**N**	**O**	**O**	**N**	**O**	**OE1**	**NE2**	**O**	**N**
**N**	2,05	-	-	-	1,98	1,88	2,2	2,03	2,05	-	2,06	2,10	-	2,06	2,0675	-	2,26	1,9
**O1**	-	-	-	2,1	-	-	-	1,95	-	-	-	-	2,2	-	-	-	-	-
**O2**	2,3	2,11	1,76	-	1,88	1,70	2,08	1,99	1,78	2,08	1,68	1,83	2,05	1,93	1,64	2,29	-	2,16
**O3**	-	2,21	-	-	1,65	1,78	-	1,79	1,84	-	1,87	1,88	-	1,79	1,765	2,07	-	-
**O4**	-	-	-	-	1,65	-	-	2,02	1,59	-	-	-	-	-	-	1,78	-	-
**O5**	-	-	-	-	-	-	-	-	-	-	-	-	-	-	-	-	-	-

Table S10 H-bonds lengths [Å] between chitosan and collagen amino acid atoms for **HD=1 and DD=1**. The numbering of atoms is the same as in Table. S1 and Figure S1.

	**ARG**	**ARG**	**ARG**	**ARG**	**GLU**	**GLU**	**GLU**	**HYP**	**HYP**	**PRO**	**PRO**	**GLY**	**ALA**	**ALA**	**GLN**	**GLN**	**GLN**	**GLN**
	**N**	**NH1**	**NH2**	**NE**	**OE1**	**OE2**	**N**	**OD1**	**O**	**N**	**O**	**O**	**N**	**O**	**OE1**	**NE2**	**O**	**N**
**N**	2,09	1,99	-	-	1,92	2,22	2,16	2,08	2,04	-	-	2,03	2,07	1,96	2,16	2,18	1,89	-
**O1**	-	-	-	-	-	-	-	-	-	-	-	-	-	-	-	1,99	-	-
**O2**	2,38	1,79	-	1,85	1,65	1,82	1,86	1,86	1,86	-	-	1,86	2,07	1,93	-	-	1,72	2,16
**O3**	1,91	2,11	2,18	1,86	1,74	1,64	-	1,93	1,92	-	-	1,80	2,03	2,11	1,72	-	-	-
**O4**	-	-	-	-	-	-	-	1,87	2,08	-	-	-	-	-	-	-	-	-
**O5**	-	-	-	-	-	-	-	-	-	-	-	-	-	-	-	-	-	-

Table S11 H-bonds lengths [Å] between chitosan and collagen amino acid atoms for **HD=0 and DD=0.125**. The numbering of atoms is the same as in Table. S1 and Figure S1.

	**ARG**	**ARG**	**ARG**	**ARG**	**GLU**	**GLU**	**GLU**	**HYP**	**HYP**	**HYP**	**PRO**	**PRO**	**GLY**	**ALA**	**ALA**	**GLN**	**GLN**	**GLN**	**GLN**
	**N**	**NH1**	**NH2**	**NE**	**OE1**	**OE2**	**N**	**OD1**	**O**	**N**	**N**	**O**	**O**	**N**	**O**	**OE1**	**NE2**	**O**	**N**
**N**	-	-	-	-	-	2.00	-	-	-	-	-	2,16	2,12	-	-	2,08	-	-	-
**O1**	-	2,16	2,27	-	-	-	-	-	-	-	-	-	-	2,43	-	-	-	-	-
**O2**	2,02	-	1,97	2,29	1,79	1,69	2,3	-	-	-	2,03	1,88	1,81	2,05	1,85	1,88	2,06	1,96	2,19
**O3**	-	1,94	1,96	1,88	-	1,79	2.00	-	-	-	2,01	1,76	1,82	2,07	1,95	1,95	1,90	1,66	2,22

	ARG	ARG	ARG	ARG	GLU	GLU	GLU	HYP	HYP	HYP	PRO	PRO	GLY	ALA	ALA	GLN	GLN	GLN	GLN
	**N**	**NH1**	**NH2**	**NE**	**OE1**	**OE2**	**N**	**OD1**	**O**	**N**	**N**	**O**	**O**	**N**	**O**	**OE1**	**NE2**	**O**	**N**
**O4**	-	1,97	-	-	1,71	-	-	-	-	-	-	-	2,04	-	1,88	-	-	-	-
**O5**	1,99	1,94	-	-	-	-	2,12	-	-	-	1,99	-	-	2,07	-	-	2,00	-	2,29

Table S12 H-bonds lengths [Å] between chitosan and collagen amino acid atoms for **HD=0.42 and DD=0.5**. The numbering of atoms is the same as in Table. S1 and Figure S1.

	ARG	ARG	ARG	ARG	GLU	GLU	GLU	HYP	HYP	PRO	PRO	GLY	ALA	ALA	GLN	GLN	GLN	GLN
	**N**	**NH1**	**NH2**	**NE**	**OE1**	**OE2**	**N**	**OD1**	**O**	**N**	**O**	**O**	**N**	**O**	**OE1**	**NE2**	**O**	**N**
**N**	-	-	-	-	1,98	2,21	-	2,13	2,07	-	1,95	2,02	-	2,02	1,96	-	2,39	-
**O1**	-	2,39	-	-	-	-	-	2,11	-	-	-	-	1,97	-	-	-	-	-
**O2**	2,18	2,01	2,01	-	1,705	-	2,18	1,88	1,92	2,09	1,8	1,85	2,06	1,85	1,74	2,03	1,83	2,01
**O3**	-	2,38	2,04	-	1,74	1,83	2,23	1,95	1,87	1,92	1,71	1,86	2,05	-	-	-	1,8	2,42
**O4**	-	-	-	-	1,85	2,08	-	1,94	1,81	-	1,87	1,65	-	-	-	2,07	-	-
**O5**	-	1,89	1,87	1,95	-	-	2,02	1,80	-	-	-	-	-	-	-	-	-	-

Table S13 H-bonds lengths [Å] between chitosan and collagen amino acid atoms for **HD=1 and DD=0.125**. The numbering of atoms is the same as in Table. S1 and Figure S1.

	ARG	ARG	ARG	ARG	GLU	GLU	GLU	HYP	HYP	PRO	PRO	GLY	GLY	ALA	ALA	GLN	GLN	GLN	GLN
	**N**	**NH1**	**NH2**	**NE**	**OE1**	**OE2**	**N**	**OD1**	**O**	**N**	**O**	**N**	**O**	**N**	**O**	**OE1**	**NE2**	**O**	**N**
**N**	-	-	-	-	2,19	2,3	-	2,08	2,24	-	-	-	2,23	-	2,27	2,2	-	2,1	-
**O1**	-	-	-	-	-	-	-	1,91	-	-	-	-	-	-	-	-	-	-	-
**O2**	-	1,9	2	1,77	1,96	1,73	2,15	1,91	1,90	-	-	1,92	1,86	2,01	1,77	2,2	1,99	2,05	2,1
**O3**	-	2,09	-	-	1,67	1,78	-	1,86	1,85	-	-	-	1,82	2,05	2,01	1,83	-	1,9	2,01
**O4**	-	2,26	-	-	1,73	-	-	1,75	1,74	-	-	-	1,74	-	-	-	-	-	-
**O5**	-	1,92	-	2,19	-	-	1,98	1,82	-	-	-	-	-	2,085	-	-	2,11	-	2,05

Table S14 Energies of H-bonds [kJ/mol] between chitosan and given collagen amino acid residue atom for **HD=0 and DD=1**. The numbering of atoms is the same as in Table. S1 and Figure S1.

	ARG	ARG	ARG	ARG	GLU	GLU	GLU	HYP	HYP	PRO	PRO	GLY	ALA	ALA	GLN	GLN	GLN	GLN
	**N**	**NH1**	**NH2**	**NE**	**OE1**	**OE2**	**N**	**OD1**	**O**	**N**	**O**	**O**	**N**	**O**	**OE1**	**NE2**	**O**	**N**
**N**	-	21,1	-	12,1	18,5	15,5	-	-	-	-	19,6	18,1	21,9	19,4	15,225	-	16,5	-
**O1**	-	-	-	-	-	-	-	-	-	-	-	-	-	-	-	-	-	-
**O2**	-	15,1	-	-	22,7	23,5	17,5	-	-	-	21,1	23,0	25	21,8	18,0	8,65	19,15	25
**O3**	-	-	-	-	24,6	22,3	-	-	-	-	20,0	20,5	16,5	24,4	21,9	21,6	24,4	9,38
**O4**	-	-	-	-	21,5	-	-	-	-	-	23,8	22,7	-	-	-	-	-	-
**O5**	-	-	-	-	-	-	-	-	-	-	-	-	-	-	-	-	-	-

Table S15 Energies of H-bonds [kJ/mol] between chitosan and given collagen amino acid residue atom for **HD=0.42 and DD=1**. The numbering of atoms is the same as in Table. S1 and Figure S1.

	ARG	ARG	ARG	ARG	GLU	GLU	GLU	HYP	HYP	PRO	PRO	GLY	ALA	ALA	GLN	GLN	GLN	GLN
	**N**	**NH1**	**NH2**	**NE**	**OE1**	**OE2**	**N**	**OD1**	**O**	**N**	**O**	**O**	**N**	**O**	**16,3**	**-**	**17,1**	**21,5**
**N**	25	-	-	-	17,9	22,9	9,3	18,1	14,6	-	18,9	17,7	-	19,0	-	-	-	-
**O1**	-	-	-	17,8	-	-	-	24,4	-	-	-	-	17,78	-	25	11,8	-	18,6
**O2**	15,1	15,3	22,9	-	23,9	23	22,88	20,2	20,3	7,8	21,8	21,9	22,8	21,4	21,59	18,0	-	-
**O3**	-	15,6	-	-	23,3	22,9	-	22,8	21, 6	-	18,8	20,2	-	18,5	-	25	-	-
**O4**	-	-	-	-	23,8	-	-	19,2	25	-	-	-	-	-	-	-	-	-
**O5**	-	-	-	-	-	-	-	-	-	-	-	-	-	-	16,3	-	17,1	21,5

Table S16 Energies of H-bonds [kJ/mol] between chitosan and given collagen amino acid residue atom for **HD=1 and DD=1**.

	ARG	ARG	ARG	ARG	GLU	GLU	GLU	HYP	HYP	PRO	PRO	GLY	ALA	ALA	GLN	GLN	GLN	GLN
	**N**	**NH1**	**NH2**	**NE**	**OE1**	**OE2**	**N**	**OD1**	**O**	**N**	**O**	**O**	**N**	**O**	**OE1**	**NE2**	**O**	**N**
**N**	19,9	24,4	-	-	17,6	13,3	12,8	15,5	16,6	-	-	20,2	7,3	15,2	15,7	8,6	23,1	-
**O1**	-	-	-	-	-	-	-	-	-	-	-	-	-	-	-	21,5	-	-
**O2**	7,18	18,6	-	21,1	23,6	22,7	13,5	22,2	20,7	-	-	20,3	17,6	16,8	-	-	22,7	17,4
**O3**	25	22,0	10,8	23,44	23,3	25	-	21,5	20,1	-	-	23,0	21,2	19,5	24,1	-	-	-

	**ARG**	**ARG**	**ARG**	**ARG**	**GLU**	**GLU**	**GLU**	**HYP**	**HYP**	**PRO**	**PRO**	**GLY**	**ALA**	**ALA**	**GLN**	**GLN**	**GLN**	**GLN**
	**N**	**NH1**	**NH2**	**NE**	**OE1**	**OE2**	**N**	**OD1**	**O**	**N**	**O**	**O**	**N**	**O**	**OE1**	**NE2**	**O**	**N**
**O4**	-	-	-	-	-	-	-	24,2	21,9	-	-	-	-	-	-	-	-	-
**O5**	-	-	-	-	-	-	-	-	-	-	-	-	-	-	-	-	-	-

Table S17 Energies of H-bonds [kJ/mol] between chitosan and given collagen amino acid residue atom for HD=0 and DD=0.125.

	**ARG**	**ARG**	**ARG**	**ARG**	**GLU**	**GLU**	**GLU**	**HYP**	**HYP**	**PRO**	**PRO**	**GLY**	**ALA**	**ALA**	**GLN**	**GLN**	**GLN**	**GLN**
	**N**	**NH1**	**NH2**	**NE**	**OE1**	**OE2**	**N**	**OD1**	**O**	**N**	**O**	**O**	**N**	**O**	**OE1**	**NE2**	**O**	**N**
**N**	-	-	-	-	-	18,6	-	-	-	-	18,22	16,6	-	-	16,9	-	-	-
**O1**	-	12,3	11,8	-	-	-	-	-	-	-	-	-	7,9	-	-	-	-	-
**O2**	17,5	-	19,9	10,6	24,2	25	14,8	-	-	15,9	20,2	22,4	21,1	22,9	18,4	22,1	21,6	16,5
**O3**	-	22,5	19,0	17,2	-	23,7	23,8	-	-	15,7	22,7	22,4	20,3	22,6	10,6	21,2	24,4	15,2
**O4**	-	21,1	-	-	23,4	-	-	-	-	-	-	13,5	-	21,1	-	-	-	-
**O5**	25	19,8	-	-	-	-	18,6	-	-	15,83	-	-	20,3	-	-	18,1	-	13,6

Table S18 Energies of H-bonds [kJ/mol] between chitosan and given collagen amino acid residue atom for HD=0.42 and DD=0.5.

	**ARG**	**ARG**	**ARG**	**ARG**	**GLU**	**GLU**	**GLU**	**HYP**	**HYP**	**PRO**	**PRO**	**GLY**	**ALA**	**ALA**	**GLN**	**GLN**	**GLN**	**GLN**
	**N**	**NH1**	**NH2**	**NE**	**OE1**	**OE2**	**N**	**OD1**	**O**	**N**	**O**	**O**	**N**	**O**	**OE1**	**NE2**	**O**	**N**
N	-	-	-	-	14,7	10,9	-	13,6	14,8	-	16,4	16,3	-	17,1	18,4	-	8,97	-
O1	-	9,7	-	-	-	-	-	16,2	-	-	-	-	14,2	-	-	-	-	-
O2	17,3	15,1	19,2	-	24,0	-	16,9	20,1	19,4	7,6	21,9	23,3	17,4	23,4	24,4	25	25	18,3
O3	-	9,2	17	-	23,4	21,8	18,6	20,6	21,2	14,5	18,7	21,8	20,5	-	-	-	21,9	7,4
O4	-	-	-	-	22,9	17,2	-	23,6	20,1	-	21,9	21,9	-	-	-	17,8	-	-
O5	-	21,0	20,0	19,6	-	-	20,7	23,1	-	-	-	-	-	-	-	-	-	-

Table S19 Energies of H-bonds [kJ/mol] between chitosan and given collagen amino acid residue atom for **HD=1 and DD=0.125**.

	ARG	ARG	ARG	ARG	GLU	GLU	GLU	HYP	HYP	HYP	PRO	PRO	GLY	ALA	ALA	GLN	GLN	GLN	GLN
	N	NH1	NH2	NE	OE1	OE2	N	O1	OD1	O	N	O	O	N	O	OE1	NE2	O	N
**N**	-	-	-	-	10,5	7,3	-	17,9	14,5	12,8	-	-	13,8	-	11,7	6,9	-	25	-
**O1**	-	-	-	-	-	-	-	16,7	13,1	-	-	-	-	-	-	-	-	-	-
**O2**	-	22,3	21,9	25	14,5	23,7	15,4	21,1	22,1	19,5	-	-	21,2	22,0	22,5	10,8	19,2	12,765	15,9
**O3**	-	14,6	-	-	25	23,0	-	21,8	22,0	21,5	-	-	21,5	18,5	12,5	16,6	-	25	23,3
**O4**	-	12,0	-	-	23,5	-	-	24,3	23,6	24,7	-	-	25	-	-	-	-	-	-
**O5**	-	24,2	-	10,8	-	-	18,2	21,5	21,8	-	-	-	-	19,9	-	-	15,1	-	23,3

Table S20 The H-bonds acceptor-donor frequency analysis. Values in cells represent how often chitosan in a given interaction plays the role of acceptor for HD=0 and DD=1.

	ARG	ARG	ARG	ARG	GLU	GLU	GLU	HYP	HYP	PRO	PRO	GLY	ALA	ALA	GLN	GLN	GLN	GLN
	N	NH1	NH2	NE	OE1	OE2	N	OD1	O	N	O	O	N	O	OE1	NE2	O	N
**N**	-	100%	-	100%	0%	0%	-	-	-	-	0%	0%	100%	0%	0%	-	0%	-
**O1**	-	-	-	-	-	-	-	-	-	-	-	-	-	-	-	-	-	-
**O2**	-	100%	-	-	0%	0%	100%	-	-	-	0%	0%	100%	0%	0%	100%	0%	100%
**O3**	-	-	-	-	0%	0%	-	-	-	-	0%	0%	100%	0%	0%	100%	0%	100%
**O4**	-	-	-	-	0%	-	-	-	-	-	0%	0%	-	-	-	-	-	-
**O5**	-	-	-	-	-	-	-	-	-	-	-	-	-	-	-	-	-	-

Table S21 The H-bonds acceptor-donor frequency analysis. Values in cells represent how often chitosan in a given interaction plays the role of acceptor for HD=0.42 and DD=1.

	ARG	ARG	ARG	ARG	GLU	GLU	GLU	HYP	HYP	PRO	PRO	GLY	ALA	ALA	GLN	GLN	GLN	GLN
	N	NH1	NH2	NE	OE1	OE2	N	OD1	O	N	O	O	N	O	OE1	NE2	O	N
**N**	100%	-	-	-	0%	0%	100%	0%	0%	-	0%	0%	-	0%	0%	-	0%	100%
**O1**	-	-	-	100%	-	-	-	100%	-	-	-	-	100%	-	-	-	-	-
**O2**	100%	100%	100%	-	0%	0%	100%	75%	0%	100%	0%	0%	100%	0%	0%	100%	-	100%

	ARG	ARG	ARG	ARG	GLU	GLU	GLU	HYP	HYP	PRO	PRO	GLY	ALA	ALA	GLN	GLN	GLN	GLN
	**N**	**NH1**	**NH2**	**NE**	**OE1**	**OE2**	**N**	**OD1**	**O**	**N**	**O**	**O**	**N**	**O**	**OE1**	**NE2**	**O**	**N**
**O3**	-	100%	-	-	0%	0%	-	50%	0%	-	0%	0%	-	0%	0%	100%	-	-
**O4**	-	-	-	-	0%	-	-	100%	0%	-	-	-	-	-	-	100%	-	-
**O5**	-	-	-	-	-	-	-	-	-	-	-	-	-	-	-	-	-	-

Table S22 The H-bonds acceptor-donor frequency analysis. Values in cells represent how often chitosan in a given interaction plays the role of acceptor for HD=1 and DD=1.

	ARG	ARG	ARG	ARG	GLU	GLU	GLU	HYP	HYP	PRO	PRO	GLY	ALA	ALA	GLN	GLN	GLN	GLN
	**N**	**NH1**	**NH2**	**NE**	**OE1**	**OE2**	**N**	**OD1**	**O**	**N**	**O**	**O**	**N**	**O**	**OE1**	**NE2**	**O**	**N**
**N**	100%	100%	-	-	0%	0%	100%	4%	0%	-	-	0%	100%	0%	0%	100%	0%	-
**O1**	-	-	-	-	-	-	-	-	-	-	-	-	-	-	-	100%	-	-
**O2**	100%	100%	-	100%	0%	0%	100%	57%	0%	-	-	0%	100%	0%	-	-	0%	100%
**O3**	100%	100%	100%	100%	0%	0%	-	50%	0%	-	-	0%	100%	0%	0%	-	-	-
**O4**	-	-	-	-	-	-	-	50%	0%	-	-	-	-	-	-	-	-	-
**O5**	-	-	-	-	-	-	-	-	-	-	-	-	-	-	-	-	-	-

Table S23 The H-bonds acceptor-donor frequency analysis. Values in cells represent how often chitosan in a given interaction plays the role of acceptor for HD=0 and DD=0.125.

	ARG	ARG	ARG	ARG	GLU	GLU	GLU	HYP	HYP	PRO	PRO	GLY	ALA	ALA	GLN	GLN	GLN	GLN
	**N**	**NH1**	**NH2**	**NE**	**OE1**	**OE2**	**N**	**OD1**	**O**	**N**	**O**	**O**	**N**	**O**	**OE1**	**NE2**	**O**	**N**
**N**	-	-	-	-	0%	0%	-	-	-	-	0%	0%	-	0%	0%	-	0%	-
**O1**	-	100%	100%	-	-	-	-	-	-	-	-	-	100%	-	-	-	-	-
**O2**	100%	-	100%	100%	0%	0%	100%	-	-	100%	0%	0%	100%	0%	0%	100%	0%	100%
**O3**	-	100%	100%	100%	0%	0%	100%	-	-	100%	0%	0%	100%	0%	0%	100%	0%	100%
**O4**	-	100%	-	-	0%	-	-	-	-	-	0%	0%	-	-	-	-	-	-
**O5**	100%	100%	-	-	-	-	100%	-	-	100%	-	-	100%	-	-	100%	-	100%

Table S24 The H-bonds acceptor-donor frequency analysis. Values in cells represent how often chitosan in a given interaction plays the role of acceptor for HD=0.42 and DD=0.5.

	ARG	ARG	ARG	ARG	GLU	GLU	GLU	HYP	HYP	PRO	PRO	GLY	ALA	ALA	GLN	GLN	GLN	GLN
	**N**	**NH1**	**NH2**	**NE**	**OE1**	**OE2**	**N**	**OD1**	**O**	**N**	**O**	**O**	**N**	**O**	**OE1**	**NE2**	**O**	**N**
**N**	-	-	-	-	0%	0%	-	0%	0%	-	0%	0%	-	0%	0%	-	0%	-
**O1**	-	100%	-	-	-	-	-	100%	-	-	-	-	100%	-	-	-	-	-
**O2**	100%	100%	100%	-	0%	-	100%	36%	0%	100%	0%	0%	100%	0%	0%	100%	0%	100%
**O3**	-	100%	100%	-	0%	0%	100%	47%	0%	100%	0%	0%	100%	-	-	-	0%	100%
**O4**	-	-	-	-	0%	0%	-	67%	0%	-	0%	0%	-	-	-	100%	-	-
**O5**	-	100%	100%	100%	-	-	100%	100%	-	-	-	-	-	-	-	-	-	-

Table S25 The H-bonds acceptor-donor frequency analysis. Values in cells represent how often chitosan in a given interaction plays the role of acceptor for HD=1 and DD=0.125.

	ARG	ARG	ARG	ARG	GLU	GLU	GLU	HYP	HYP	PRO	PRO	GLY	ALA	ALA	GLN	GLN	GLN	GLN
	**N**	**NH1**	**NH2**	**NE**	**OE1**	**OE2**	**N**	**OD1**	**O**	**N**	**O**	**O**	**N**	**O**	**OE1**	**NE2**	**O**	**N**
**N**	-	-	-	-	0%	0%	-	0%	0%	-	-	0%	-	0%	0%	-	0%	-
**O1**	-	-	-	-	-	-	-	100%	-	-	-	-	-	-	-	-	-	-
**O2**	-	100%	100%	100%	0%	0%	100%	65%	0%	-	-	0%	100%	0%	0%	100%	0%	100%
**O3**	-	100%	-	-	0%	0%	-	82%	0%	-	-	0%	100%	0%	0%	-	0%	100%
**O4**	-	100%	-	-	0%	-	-	75%	0%	-	-	0%	-	-	-	-	-	-
**O5**	-	100%	-	100%	-	-	100%	100%	-	-	-	-	100%	-	-	100%	-	100%
